# Peripheral myeloid-derived suppressor and T regulatory PD-1 positive cells predict response to neoadjuvant short-course radiotherapy in rectal cancer patients

**DOI:** 10.18632/oncotarget.3014

**Published:** 2015-01-21

**Authors:** Maria Napolitano, Crescenzo D'Alterio, Eleonora Cardone, Anna Maria Trotta, Biagio Pecori, Daniela Rega, Ugo Pace, Dario Scala, Giosuè Scognamiglio, Fabiana Tatangelo, Carmela Cacciapuoti, Roberto Pacelli, Paolo Delrio, Stefania Scala

**Affiliations:** ^1^ Immunology Unit, Istituto Nazionale per lo Studio e la Cura dei Tumori “Fondazione Giovanni Pascale” – I.R.C.C.S.-Naples, Italy; ^2^ Colorectal Surgery, Istituto Nazionale per lo Studio e la Cura dei Tumori “Fondazione Giovanni Pascale” – I.R.C.C.S.-Naples, Italy; ^3^ Radiotherapy, Istituto Nazionale per lo Studio e la Cura dei Tumori “Fondazione Giovanni Pascale” – I.R.C.C.S.-Naples, Italy; ^4^ Pathology, Istituto Nazionale per lo Studio e la Cura dei Tumori “Fondazione Giovanni Pascale” – I.R.C.C.S.-Naples, Italy; ^5^ Trasfusional Service, Istituto Nazionale per lo Studio e la Cura dei Tumori “Fondazione Giovanni Pascale” – I.R.C.C.S.-Naples, Italy; ^6^ Department of Advanced Biomedical Sciences, Federico II University School of Medicine, and Istituto di Biostrutture e Bioimmagini – C.N.R., Naples, Italy

**Keywords:** MDSC, Treg, radiotherapy, rectal cancer

## Abstract

Short-course preoperative radiotherapy (SC-RT) followed by total mesorectal excision (TME) is one therapeutic option for locally advanced rectal cancer (LARC) patients. Since radio-induced DNA damage may affect tumor immunogenicity, Myeloid-derived suppressor cells (MDSCs) and T regulatory cells (Tregs) were evaluated in 13 patients undergoing SC-RT and TME for LARC. Peripheral Granulocytic-MDSCs (G-MDSC) [LIN^−^/HLA-DR^−^/CD11b^+^/CD14^−^/CD15^+^/CD33^+^], Monocytic (M-MDSC) [CD14^+^/HLA-DR^−/low^CD11b^+^/CD33^+^] and Tregs [CD4^+^/CD25^hi+^/FOXP3^+^- CTLA-4/PD1] basal value was significantly higher in LARC patients compared to healthy donors (HD). Peripheral MDSC and Tregs were evaluated at time 0 (T0), after 2 and 5 weeks (T2-T5) from radiotherapy; before surgery (T8) and 6–12 months after surgery (T9, T10). G-MDSC decreased at T5 and further at T8 while M-MDSC cells decreased at T5; Tregs reached the lowest value at T5. LARC poor responder patients displayed a major decrease in M-MDSC after SC-RT and an increase of Treg-PD-1. In this pilot study MDSCs and Tregs decrease during the SC-RT treatment could represent a biomarker of response in LARC patients. Further studies are needed to confirm that the deepest M-MDSC reduction and increase in Treg-PD1 cells within 5–8 weeks from the beginning of treatment could discriminate LARC patients poor responding to SC-RT.

## INTRODUCTION

Microenvironment (ME) surrounding cancer cells has been shown to be profoundly involved in the biological behaviour of tumours. Through an active humoral and physical cross talk ME can determine invasion, ability to metastasize, immunogenicity of cancer and response to anticancer therapies including radiation therapy (RT). The effects of radiotherapy reported on tumor cells are conflicting, and the general belief on immunity is that different microenvironments and diverse delivery modalities may induce activation or inhibition of immune response and the size of fraction is one of the variables involved in such a dualism [1–3].

Colorectal cancer is the most commonly diagnosed cancer in Europe and a leading cause of cancer death worldwide [4]. Approximately thirty percent of colorectal adenocarcinomas arise in the rectum [4]. The adoption of total mesorectal excision (TME) and the transition from post-operative to preoperative treatment have improved the management of locally advanced rectal cancer (LARC), resulting in a significant decline of the local recurrence rate [5]. Nevertheless, approximately one third of patients with LARC will develop distant metastases [5]. The management of the middle and low rectum LARC includes neoadjuvant radiotherapy (NRT) [6] that has been shown to be less toxic and more effective in improving local control than postoperative RT [7]. Two most popular NRT schedule are actually preferred: the long-course RT (45–50.4 Gy in 25–28 1.8 Gy fractions delivered in about 5 weeks coupled with fluoropirimidine followed by surgery 4–8 weeks later) and short-course radiation therapy (SC-RT) (25 Gy in 5 fractions in a week with surgery performed within a week from the end of SC-RT) [8–10]. More recently delayed surgery after SC-RT has been proposed [11, 12] also for high risk LARC patients in association with chemotherapy [12].

Colorectal cancer ME exhibits immune/inflammatory infiltrates with up-regulation of characteristic ‘inflammatory signature’ genes [13]. Although infiltrating CD4^+^ Th1 cells and CD8^+^ cytotoxic T cells sign a positive prognosis in colorectal cancer [14], the immunosuppressive regulatory T cells and myeloid cells promote tumorigenesis [13]. Myeloid-derived suppressor cells (MDSCs) are a heterogeneous population composing of cells at several stages of differentiation of the myeloid lineage, accumulate in the blood, lymph nodes, bone marrow, and tumor sites in patients and experimental animals with cancer, and are capable of inhibiting both innate and adaptive immune responses [15, 16]. MDSCs influence both innate and adaptive immune responses through: depletion of nutrients required by lymphocytes - specifically, l-arginine depletion through ARG1-dependent consumption and l-cysteine deprivation via its consumption and sequestration [17], generation of oxidative stress, which is caused by the production of ROS and reactive nitrogen species by MDSCs [15], impairment of lymphocyte trafficking and viability [15], activation and expansion of Tregs populations [15]. MDSCs promote the clonal expansion of antigen-specific natural Tregs and also induce the conversion of naive CD4^+^ T cells into induced Treg cells. The mechanisms are not completely understood, but may involve cell-to-cell contact [15], the production of soluble factors (such as IFNγ, IL-10 and TGFβ) and possibly the expression of ARG1 by MDSCs [15]. Tregs control immune responses by suppressing conventional effector T lymphocytes, NK, DCs or macrophages through different mechanisms [18]. They are produced during T-cell development in the thymus or are generated in the periphery from naive CD4^+^ T lymphocytes. Compelling studies in mice and human have demonstrated that many cancers can induce the proliferation of Tregs and/or promote their generation from naive T cells, resulting in the accumulation of these cells in the tumor beds and in the periphery [19]. Importantly, the elimination and/or functional inactivation of tumor-induced Tregs can promote antitumor immunity and enhance the efficacy of immunotherapy [20]. With the intent to define predictive biomarkers of response a pilot study was conducted on peripheral MDSC and Tregs in LARC patients undergoing SC-RT.

## RESULTS

### Circulating granulocyte/monocyte-MDSC and Tregs decreased in LARC patients subjected to SC-RT

**Figure 1:**
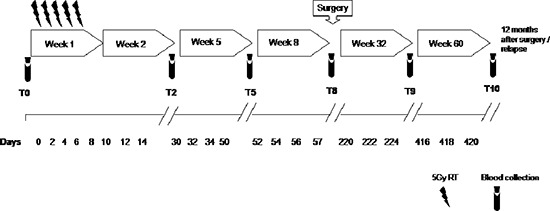
Study schedule Peripheral blood was collected at T0, pre-radiotherapy; T2, after 2 weeks from the beginning of radiotherapy; T5, after 5 weeks from the beginning of radiotherapy; T8, pre-surgery, after 8 weeks from the beginning of radiotherapy; T9, 6 months after surgery; T10, 12 months after surgery.

**Figure 2:**
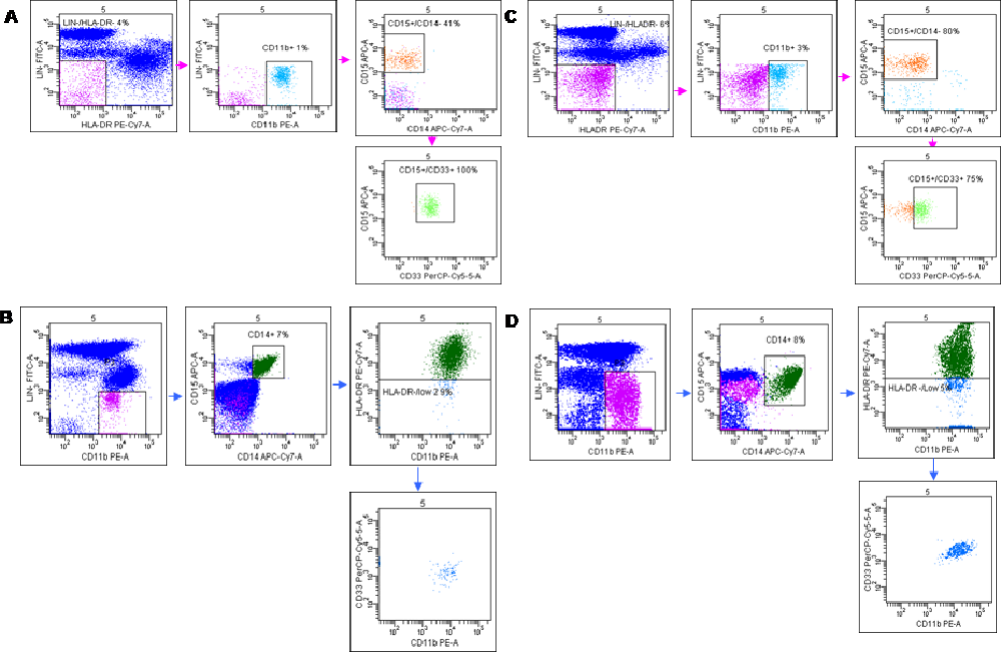
Flow cytometry analysis of Granulocytic (G-MDSc LIN-/HLA-DR−/CD11b+/CD14−/CD15+/CD33+) and Monocytic (M-MDSc CD14+/HLADR−/low/CD11b+CD33+) Peripheral blood from healthy donors **(A, B)** and LARC patients **(C, D)** were stained for the G-MDSC markers Lineage 1 (CD3/CD14/CD19/CD20/CD56), HLA-DR, CD33, CD11b, and CD15 **(A, C)** and the M-MDSC markers CD14 and HLA-DR **(B, D)**. Representative dot plot is shown. Gates were set based on isotype controls. Numbers represent the percentages from the parental populations gated. The gating strategy used to analyze the samples is illustrated. Acquired cells were first gated (Pe-Cy7/Fitc subset) based on the expression of LIN1 and HLA-DR. Within this population the fraction of cells G-MDSc expressing both CD11b, CD14, CD33 and CD15 was determined. Therefore, G-MDSC were defined as LIN^−^/HLA-DR^−/low^/CD11b^+^/CD14^−^/CD15^+^/CD33^+^ cells (**A** and **C**). Acquired cells were first gated (Pe/Fitc subset) based on the expression of LIN1 and CD11b. Within this population the fraction of cells M-MDSc expressing both CD14, CD15, HLA-DR, and CD33 was determined. Therefore, M-MDSC was defined as CD14^+^/HLA-DR^−/low^/CD11b^+^/CD33^+^cells (**B** and **C**). MDSCs percentage was calculated as percentage of total nucleated cells in whole blood samples.

**Figure 3:**
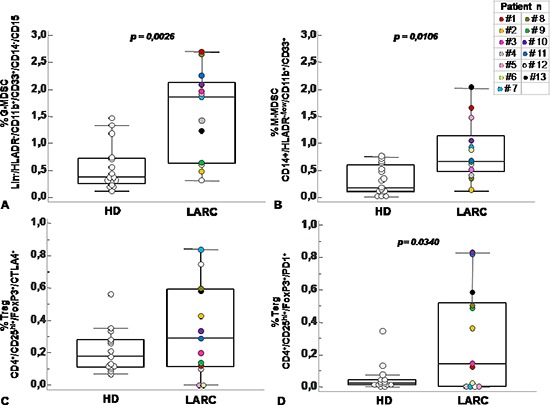
Circulating MDSC and Treg cells increased in peripheral blood of patients with rectal cancer Percentage of G-MDSC (Lin^−^/HLADR^−^/CD11b^+^/CD14^−^/CD15^+^/CD33^+^) **(A)** M-MDSC (CD14^+^/HLADR^−/low^/CD11b^+^/CD33^+^) **(B)** and Tregs subpopulations (CD4^+^/CD25^hi+^/FoxP3^+^/CTLA4^+^ and CD4^+^/CD25^hi+^/FoxP3^+^/PD1^+^) **(C–D)** calculated as percentage of total leukocyte and total lymphocyte in rectal cancer patients (RC) at baseline level (T0) and healthy donor (HD). Data are presented as dot plots, with a black line at the population median. Statistical significance was determined by Mann-Whitney U nonparametric test *p* < 0,05.

**Figure 4:**
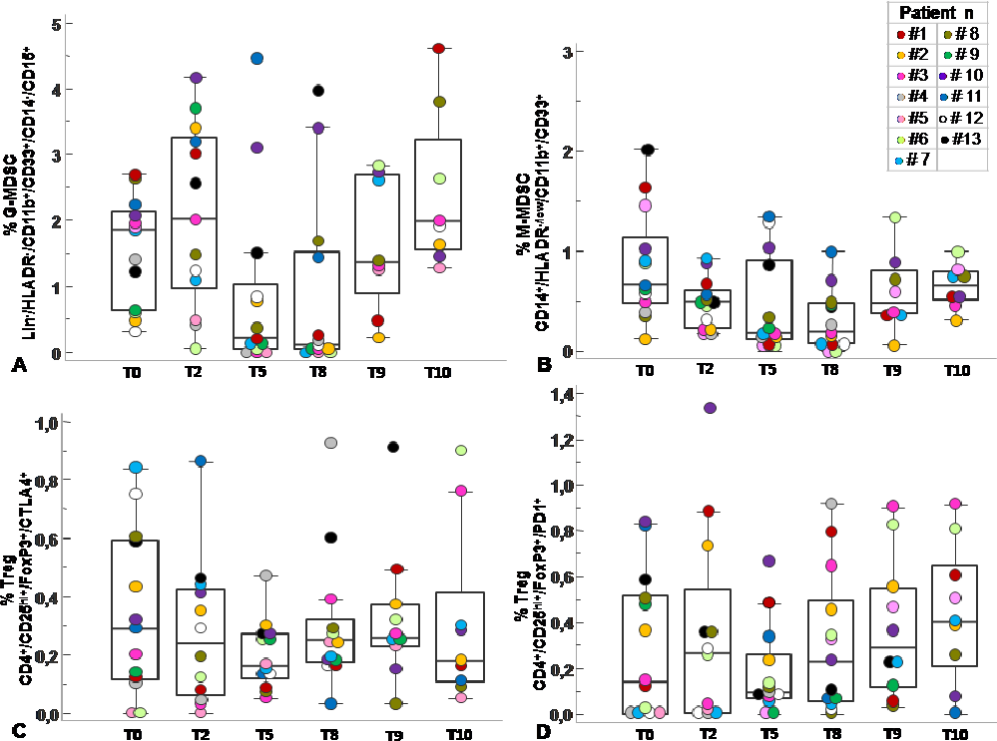
Circulating MDSC and Tregs course over SC-RT treatment Percentage of G-MDSC (Lin^−^/HLADR^−^/CD11b^+^/CD14^−^/CD15^+^CD33^+^) **(A)** M-MDSC (CD14^+^/HLADR^−/low^/CD11b^+^/CD33^+^) **(B)** and Tregs subpopulations (CD4^+^/CD25^hi+^/FoxP3^+^/CTLA4^+^ and CD4^+^/CD25^hi+^/FoxP3^+^/PD1^+^) **(C–D)** was calculated as percentage of total leukocyte and total lymphocyte respectively at T0, pre-radiotherapy; T2, after 2 weeks from the beginning of radiotherapy; T5, after 5 weeks from the beginning of radiotherapy; T8, pre-surgery, after 8 weeks from the beginning of radiotherapy; T9, 6 months after surgery; T10, 12 months after surgery. Data are presented as dot plots, with a black line at the population median. *p* < 0,05 value.

Previous studies have described significant increase in circulating Lin^−^/HLADR^−^/CD11b^+^CD33^+^ MDSCs in the peripheral blood of patients with advanced cancer including colon cancer [21]. With the intent to identify peripheral pattern of immune modulation in response to treatment, a pilot study was conducted on 13 LARC patients subjected to neo adjuvant SC-RT (5 fractions of 5Gy for 5 days) and then surgery (Table 1). MDSC and Treg cells were evaluated at time 0 (T0), after two weeks from the beginning of RT (T2), after 5 weeks from the beginning of radiotherapy (T5); before surgery (T8),6 (T9) and 12 (T10) months after surgery (Figure 1). In Figure 2 it is shown a representative flow cytometry analysis on peripheral blood from healthy individual (Figure 2A–2B) and a LARC patient (Figure 2C–2D) for G-MDSC (LIN^−^/HLA-DR^−^/CD11b^+^/CD14^−^/CD15^+^/CD33^+^and M-MDSC (CD14^+^/HLA-DR^−/low^/CD11b^+^/CD33^+^). As previously reported [21], a significantly higher number of circulating G-MDSCs (mean 1,85% vs 0,56%; *p* = 0.0026) and M-MDSC (mean 0,66% vs 0,31%; *p* = 0.0106) was detected in LARC patients at time 0 compared to healthy donors (Figure 3A–3B); also Tregs (CD4^+^/CD25^hi+^/FoxP3^+^/CTLA4^+^ and CD4^+^/CD25^hi+^/FoxP3^+^/PD1^+^) were higher in LARC patients compared to healthy donors (mean 0.18% vs 0.29% and mean 0.03% vs. 0.14%; *p* = 0.0340) (Figure 3C–3D). The MDSCs and Tregs course over treatment was then evaluated in 13 patients undergone to SC-RT followed by surgery. G-MDSC decreased at T5 and further at T8 while M-MDSC cells decreased at T5 being stable up to T8 (Figure 4A–4B); Tregs reached the lowest value at five weeks after the beginning of SC-RT (Figure 4C–4D). Tregs variations are not related to CD4 total population changes at the indicated time points (Supplementary Figure 1).

**Table 1:** Patients characteristics

Patient ID	Age, y	Gender	Postoperative AJCC stage	Tumor invasion	Lymph nodal status	CRM^§^	TRG*	Tumor Budding	Current Status°
1	81	F	I	yT1	NX	Positive	2	high	NED
2	66	M	I	yT2	N0	negative	2	high	NED
3	77	F	II	yT3	N0	negative	2	high	NED
4	81	M	II	yT3	N0	negative	3	low	DOD
5	48	M	I	yT2	N0	negative	2	high	AWD
6	74	M	IIIb	yT2	N2b	negative	3	high	NED
7	69	M	I	yT2	NX	negative	1	absent	NED
8	59	M	I	yT2	N0	negative	1	absent	NED
9	68	F	I	yTis	N0	negative	0	absent	NED
10	71	M	I	yT1	N0	negative	1	absent	NED
11	66	M	I	yT1	N0	negative	1	high	NED
12	71	M	I	yT2	N0	negative	1	absent	NED
13	44	M	I	yT1	N0	negative	2	low	NED

* Mandard system modified by Ryan TRG 0–1, Good Responder; TRG 2–3, Poor responder.

° AWD, alive with disease; DOD, dead of disease; NED, not evidence of disease

§ CRM, circumferential resection margin

### Circulating MDSCs and Tregs correlated with the tumor response to SC-RT

**Figure 5:**
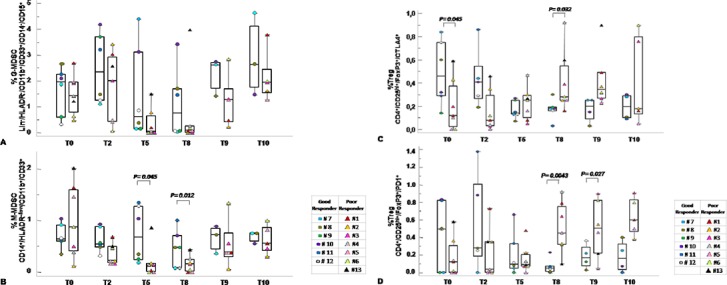
Circulating MDSC and Tregs correlated with tumor response to SC-RT Percentage of G-MDSC (Lin^−^/HLADR^−^/CD11b^+^/CD14^−^/CD15^+^/CD33^+^) **(A)** and M-MDSC (CD14^+^/HLADR^−/low^/CD11b^+^/CD33^+^ **(B)** and Tregs CD4^+^/CD25^hi+^/FOXP3^+^/CTLA-4^+^ **(C)** and CD4^+^/CD25^hi+^/FOXP3^+^/PD-1^+^ **(D)** distribution within TRG 0–1 versus TRG 2–3 at the indicated time points. MDSCs and Treg cells were calculated as percentage of total leukocyte and total lymphocyte respectively. Data are presented as dot plots, with a black line at the population median. *p* < 0,05 value.

Patients were grouped according to the pathological evaluation of Tumor Regression Grade (TRG) [22] as good (TRG 0–1) and poor responders (TRG 2–3) to SC-RT. 6/13 patients were classified as good responder and 7/13 were defined as poor-responder to SC-RT treatment. As shown in Figure 5A–5B G-MDSCs decreased in poor responder patients at T5 and T8 compared to good responder patients. M-MDSCs were significantly lower in poor responder patients at 5 weeks from the beginning of radiotherapy T5 (*p* = 0,045) and before surgery T8 (*p* = 0,012). Although limited conclusions can be drown due to the number of patients, the peripheral value of Tregs and MDSC, mainly M-MDSC at 5 and then 8 weeks post RT treatment, significantly correlate with the pathological response to RT. Functional Tregs suppression derive from the balance of costimulatory and coinhibitory molecules that are crucial for maintaining self-tolerance and modulating the immune responses in peripheral tissues [23]. As shown in Figure 5C, CD4^+^/CD25^hi+^/FOXP3^+^/CTLA-4^+^ appeared significantly higher at T0 in good responders (*p* = 0.045) with a decrease up to T5. Of note, poor responder patients displayed lower basal level compared to good responders; then CD4^+^/CD25^hi+^/FOXP3^+^/CTLA-4^+^ subpopulation increased at T5 and T8 (*p* = 0.032). In Figure 5D Tregs CD4^+^/CD25^hi+^/FOXP3^+^/PD-1^+^ clearly decreased in good responder patients with a minimum at T8. In poor-responder patients the CD4^+^/CD25^hi+^/FOXP3^+^/PD-1^+^ increased being significantly different at T8 (*p* = 0.0043) and T9 (*p* = 0.027). Thus in poor responder SC-RT patients the peripheral detection of Tregs subpopulation with high suppressive capability (CD4^+^/CD25^hi+^/FOXP3^+^/PD-1^+^) increased from T8 suggesting a resistance to SC-RT.

### MDSCs and tregs cells infiltrated post treatment rectal cancer

**Figure 6:**
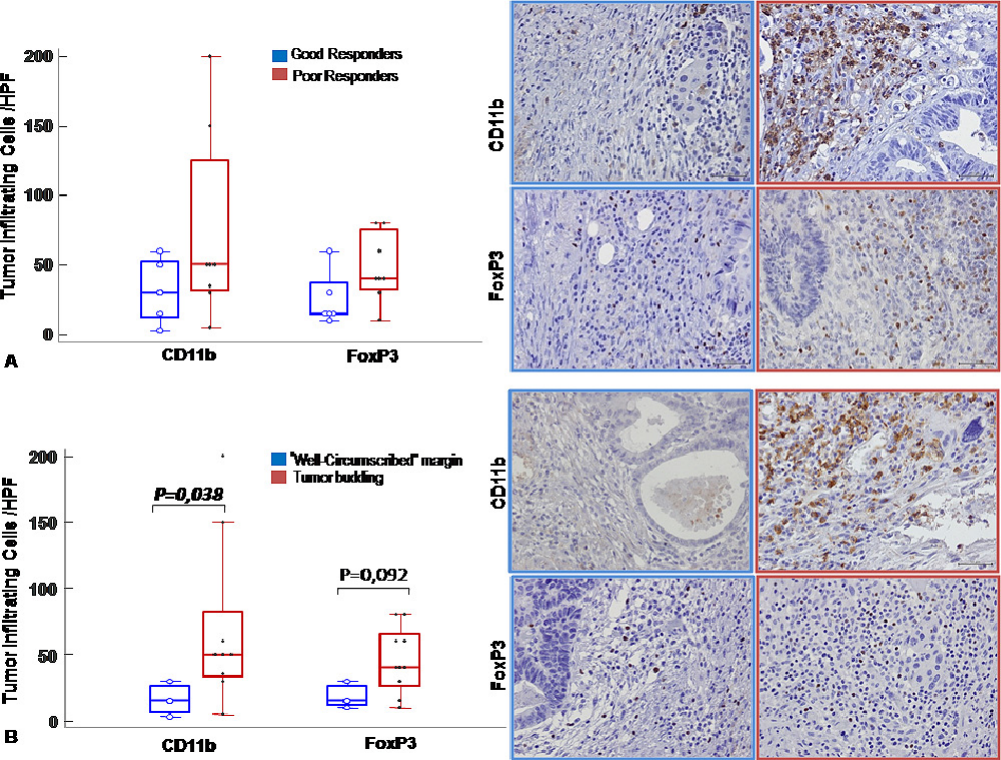
MDSC and Tregs cells infiltrated post treatment rectal cancer CD11b and FoxP3 immunohistochemical staining were conducted to reveal respectively MDSCs and Tregs tumor infiltrating cells. **(A)**. *Left panel*: Number of infiltrating cells/HPF in good responder (blue line) versus poor responder (red line) patients shown as box and whisker plots: boxes extend from the 25th to the 75th percentile, with a colored line at the population; median; *p* < 0,05 value. *Right panel*: Representative tissue staining for CD11b and FoxP3 in good responder (blue line) and poor responder (red line) patients (Magnification 200X). **(B)**. *Left panel*: Number of infiltrating cells/HPF in tumors with infiltrative margin (tumor budding) (red line) and tumors with “well-circumscribed” margin (blue line) shown as box and whisker plots: boxes extend from the 25th to the 75th percentile, with a colored line at the population; median; *p* < 0,05 value. *Right panel*: Representative tissue staining for CD11b and FoxP3 in tumors with infiltrative margin tumors (tumor budding) (red line) and well-circumscribed” (blue line) margin tumors patients (Magnification 200X).

To correlate the peripheral MDSCs and Tregs with the primary tumour, CD11b [24, 25] and FOXP3 were evaluated on the surgical specimens (Supplementary Table 1). Increased staining for CD11b and FOXP3 was detected in poor responder patients compared to good responders (Figure 6A–6B; see Supplementary Figure 2) [21, 26], specifically in island of tumor budding, a rectal cancer poor prognostic factor [27] (Figure 6B). These results suggest that the peripheral evaluation of MDSCs and Tregs might correlate with the SC-RT tumor response.

## DISCUSSION

In this study, we evaluated the immunosuppressive phenotype of circulating MDSCs and Tregs in 13 LARC patients subjected to SC-RT followed by delayed surgery. In accordance with previous studies, higher MDSCs (G- and M-MDSC cells) and Tregs were detected in the peripheral blood of LARC patients at baseline compared to healthy donors [21, 23, 26]. MDSCs and Tregs decreased in the peripheral blood during and after the SC-RT, 5 and 8 weeks from the beginning of SC-RT suggesting that this reduction may represent the tumour response to SC-RT. In patients with colon cancer, MDSCs levels were reported to be higher compared to healthy donor, correlate with cancer stage, metastasis and chemotherapy response. A similar increase was also observed in the tumor tissues [21, 26]. Despite limited number, grouping the patients according to the pathological response to SC-RT displayed a significant reduction in M-MDSC in patients with a consistent residual disease (TRG 2/3) at 5 and 8 weeks post SC-RT. Conversely Tregs increased in patients with a consistent residual disease (TRG 2/3) accompanied by increased tumour infiltration with higher CTLA4 and PD1-Treg. It was reported that CTLA-4 and PD-1, although inhibitory for T effector cells, have been implicated in the formation of inducible Tregs as well as in Treg suppressive function, respectively [27, 28].

The biological responses of tumors to radiation include DNA damage, modulation of signal transduction, and alteration of the inflammatory tumor microenvironment [29]. Radiation treatment induces the inflammatory response and tumors may develop multiple resistance mechanisms that facilitate tumor relapse [30]. Inflammation mediates these effects by up-regulating the expression of CD14 and increasing signalling through the TLR4 pathway in MDSCs [31]. The mechanism of preferential accumulation of M-MDSC in tumor site is currently not clear. It is possible that the nature of chemokines produced by tumor cells is responsible for preferential migration of M-MDSC to the tumor site. Alternatively, tumor microenvironment due to hypoxia or low pH, may not support survival of G-MDSC [32]. In preclinical mouse models treated with fractionated RT an enhanced migration of DCs and CD8 T cell and immune-suppressive myeloid-derived suppressor cells inside the tumor has been shown [33]. Tregs infiltration directly correlated to an enhanced number in the peripheral blood since the Tregs infiltration is less strictly dependent by inflammation [18] and more resistant to radiation compared to other lymphocytes. Moreover, the MDSCs regulate Tregs number and function accounting for a temporal discrepancy between MDSC and Treg cells course. Targeting this population may allow enhancement of radio therapeutic benefit through immune modulation.

Therapeutic blockade of immune checkpoints has been associated with a reversal in the distribution and proportion of MDSCs [34, 35]. CTLA4, the first immune-checkpoint receptor to be clinically targeted, is expressed exclusively on T cells where it primarily regulates the amplitude of the early stages of T cell activation [23]. Although the mechanism by which CTLA-4 enhances the immunosuppressive function of Tregs is not known, Tregs specific CTLA-4 knockout or blockade significantly inhibits their ability to regulate both autoimmunity and antitumor immunity [36, 37]. In contrast to CTLA-4, the major role of PD-1 is to limit the activity of T cells in peripheral tissues at the time of an inflammatory response to infection and to limit autoimmunity [38, 39]. This translates into a major immune resistance mechanism within the tumour microenvironment [40, 41].

Thus PD-1 predominantly regulates effector T cell activity within tissue and tumours, whereas CTLA-4 predominantly regulates T cell activation. PD-1 is more broadly expressed than CTLA-4: it is induced on other activated non-T lymphocyte subsets, including B cells and natural killer (NK) cells [23], which limits their lytic activity. Although PD-1 blockade is typically viewed as enhancing the activity of effector T cells in tissues and in the tumour microenvironment, it also probably enhances NK cell activity in tumours and tissues and may also enhance antibody production either indirectly or through direct effects on PD-1^+^ B cells [23].

The result of this pilot study, limited by the patients number, shows a clear decrease in peripheral MDSCs and Tregs at 2–6 weeks after the beginning of SC-RT in LARC patients; a possible early marker of SC-RT response. Moreover, although needed to be confirmed in a larger series of patients, a concomitant reduction in M-MDSC and a relative increase in the Treg-PD-1 subpopulation was detected in LARC patients poor responder to SC-RT suggesting that this peripheral evaluation may be an early marker of response.

## MATERIALS AND METHODS

### Ethical statement

All patients and healthy donors provided written informed consent prior to blood sampling. The research protocol n. CEI/423/13 was approved by Human Ethical Committee of Institute.

### Patients

The study was conducted on fifteen healthy donors (HD) (mean age 53, range 44–65, years) and thirteen LARC patients (mean age 66, range 43–81, years) undergoing SC-RT (25 Gy in 5 fractions in a week with surgery performed within 8 weeks from the end of SC-RT) (Figure 1). All cases were classified according to American Join Committee on Cancer TNM Staging, 6th edition, 2002. According to National Comprehensive Cancer Network (NCCN) rectal cancer guideline 2015 pathological response post-treatment was measured by Mandard Tumour Regression Grade (TRG) modified by Ryan et al [22, 42]. Table 1 shows the clinical characteristics of study patients.

### Antibodies and flow cytometric analysis

Flow cytometry was performed on fresh venous blood (BD Biosciences), using a FACS Canto II 6-colour flow cytometer, daily calibrated [43] with Calibrite beads (Fitc, Pe, PerCP and APC) and Compbeads (Pe-Cy7 and APC-Cy7; Becton Dickinson, San Jose, CA, USA). For identification of circulating MDSCs: FITC-anti-Lineage 1 antibodies (CD3, CD14, CD16, CD19, CD20, CD56), PE-anti-CD11b, PercP-anti-CD33, PE-Cy7-anti-HLA-DR, APC-anti-CD15 and APC-Cy7-anti-CD14 (BD Bioscience, San Diego, CA, USA). For identification of circulating Tregs: Fitc-anti-FOXP3, Pe-anti-FOXP3, Pe-anti-CD152 (CTA-4), PercP-anti-CD45R0, PercP-anti-CD184 (CXCR4), Pe-Cy7-anti-CD25, APC-anti-CD45RA, APC-anti-CD279 (PD-1) and APC-Cy7-anti-CD4. Monoclonal antibodies were used together with the appropriate corresponding isotype controls. A minimum of 50,000 events for each sample was collected and data were analysed using Facs Diva software. Intracellular staining for Fox-P3 was performed using a commercially available kit (BD Cytofix/Cytoperm; fixation and permeabilization kit; BD Pharmingen) according to the manufacturer's instructions.

For Treg analysis from peripheral blood, the lymphocytes were gated by FSC x SSC, CD4^+^ cells, CD25-high and FOXP3 positivity plus the expression of CTLA-4 and PD-1 and Treg cells was calculated as percentage of total lymphocyte. For MDSC analysis from peripheral blood, cells were surface-stained for lineage cocktail 1, HLA-DR, CD11b, CD14, CD15, and CD33 for G-MDSc or CD14 and HLA-DR for M-MDSc. The G-MDSc cells gated for lineage-negative/HLA-DR negative, this myeloid subset assessed for CD11b^+^/CD14^−^/CD15^+^/CD33^+^ cells (Figure 2). M-MDSc cells gated for lineage-negative/CD11b^+^ cells, then this subset for CD14^+^/HLA-DR^low/−^/CD33^+^ cells (Figure 2). MDSCs percentage was calculated as percentage of total nucleated cells in whole blood samples. The healthy donors (*N* = 15) values for MDSC subpopulations: % G-MDSC mean 0,5560 ± 0,4360; % M-MDSC mean 0,3093 ± 0,2743; for Tregs subpopulations % CD4^+^/CD25^hi+^/FoxP3^+^/CTLA4^+^ mean 0,2111 ± 0,1348 and % CD4^+^/CD25^hi+^/FoxP3^+^/PD1^+^ mean 0,05443 ± 0,08799.

### Immunohistochemistry

Surgical derived tissue specimens from the study population were retrospectively analyzed on formalin-fixed and paraffin-embedded (FFPE) tissue blocks. Slides were incubated with the following primary Abs for 1 hour at RT: Rabbit monoclonal to anti-human CD11b (clone EP1345Y, dilution 1:100, Abcam, Cambridge, UK); rabbit polyclonal anti-human FOXP3 (clone Poly6238, dilution 1:100, BioLegend Inc). EnVision+ Systems-horseradish (Dako) were used for 45 min at RT as a secondary Ab and visualization was performed with a DAB + (diaminobenzidine) substrate chromogen (Dako), according to the manufacturer's instructions. Evaluation of CD11b^+^ and FOXP3^+^ was conducted. All hematoxylin and eosin (H&E)-stained slides of tumor tissue were reviewed by an expert pathologist (FT), to confirm the diagnosis. To evaluate immune cell numbers in the invasive margin and tumor core, 5 regions of interest were evaluated for each slide. These areas with high immune cell density were identified at low power (100× magnification) and immunoregulatory cells were counted in 5 consecutive high-power field (HPF) 400× magnification (0.237 mm^2^/field), using an Olympus BX51 microscope (Olympus, Tokyo, Japan). The evaluation of stained immune cells was performed in duplicate blindly by three independent observers (FT; CD and GS). Variations in the enumeration within a range of 5% were re-evaluated and a consensus decision was made. The results were expressed as the mean number of positively stained cells/HPF through 5 region of interest.

### Data analysis and statistics

Our primary objective was to compare immunephenotypes in patients with LARC (*N* = 13) and healthy controls (*N* = 15). Repeated-measures ANOVA were used for comparison between 2 groups over time. The non-parametric Mann-Whitney U test and Kruskal-Wallis test were used to evaluate the significance of the differences of the mean ranks, owing to a lack of compatibility to the normal distribution. Per-comparison two-sided *p* values less than 0.05 were considered statistically significant. Analyses were performed using MedCalc statistical software Version 12.3.0.

## SUPPLEMENTARY FIGURES AND TABLE


