# Supplementary material for: Peripheral myeloid-derived suppressor and T regulatory PD-1 positive cells predict response to neoadjuvant short-course radiotherapy in rectal cancer patients
Source: Oncotarget. 2015 Jan 21;6(10):8261–70. doi: 10.18632/oncotarget.3014 (PMC4480750; doi:10.18632/oncotarget.3014)
Supplement: Supplementary file 1 [file oncotarget-06-8261-s001.pdf]

SUPPLEMENTARY FIGURES AND TABLE

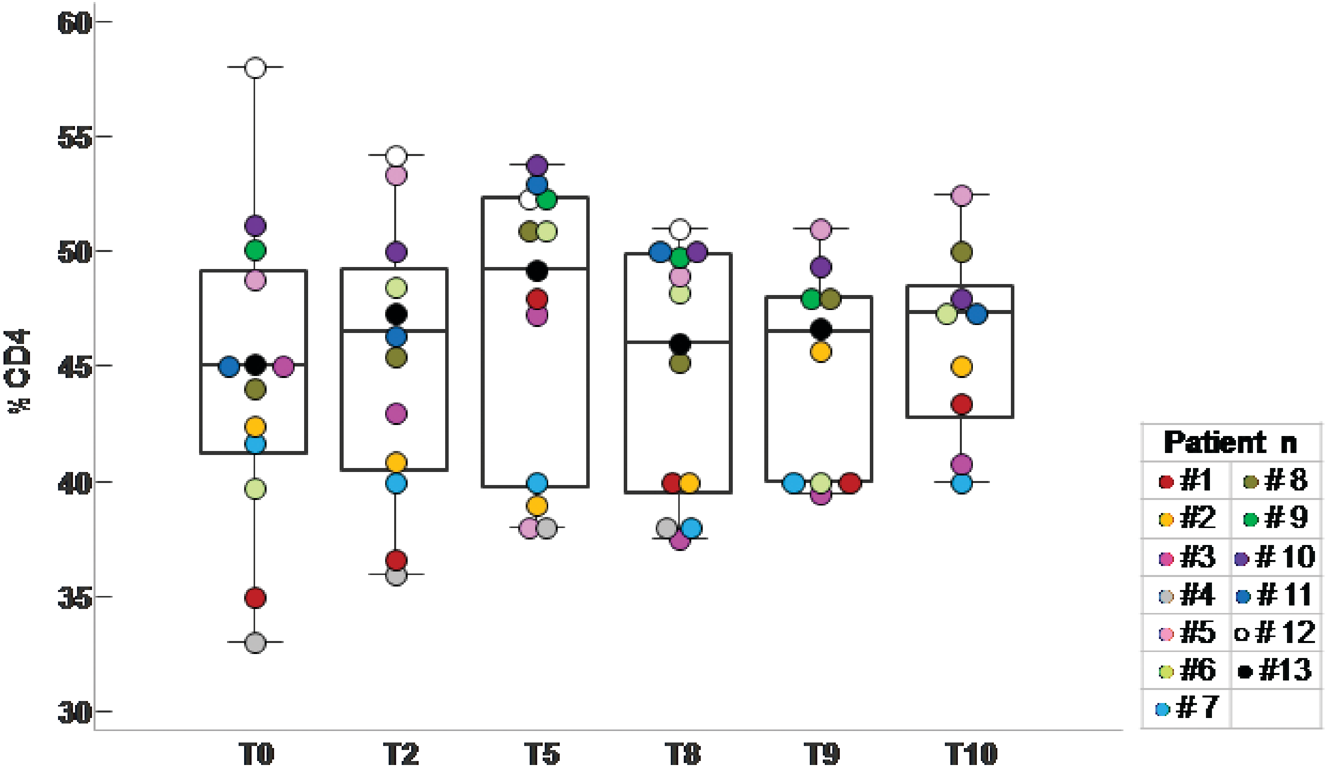

Supplementary Figure 1: CD4 in Study population. % CD4 cell variations at time points in study cohort.

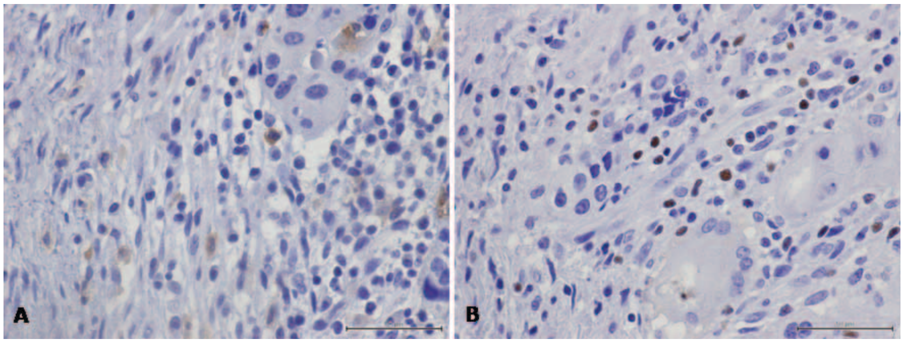

Supplementary Figure 2: Higher magnification ofMDSC and Tregs cells infiltrated post treatment rectal cancer. CD11b immunostaining with cytoplasmic localization by infiltrating myeloid cells **(A)** and FOXP3 with nuclear localization, **(B)** (400x magnification). Scale bar: 50 µm.

Supplementary Table 1: IHC staining of Tumor infiltrating MDSC and Tregs in study population

			IHC		T8 FACS				
Patient ID	TRG*	Tumor Budding	CD11b	Fox-P3	Lin-/HLADR-/CD11b+/CD33+/CD14-/CD15+	CD14^{+}/HLADR$^{-/low}$/CD11b^{+}/CD33^{+}		CD4^{+}/CD25^{hi+}/FoxP3^{+}/CTLA4^{+}	CD4^{+}/CD25^{hi+}/FoxP3^{+}/PD1^{+}
			cells/HPF		%				
1	2	high	35	40	0,26	0,05		0,16	0,79
2	2	high	50	80	0,09	0,16		0,25	0,45
3	2	high	30	30	0,08	0,19		0,39	0,64
4	3	low	200	60	0,18	0,27		0,92	0,92
5	2	high	50	10	0,00	0,00		0,25	0,32
6	3	high	5	40	0,00	0,00		0,28	0,33
7	1	absent	3	15	0,01	0,09		0,19	0,04
8	1	absent	60	15	1,69	0,49		0,30	0,00
9	0	absent	–	–	0,06	0,47		0,18	0,06
10	1	absent	30	30	3,41	0,71		0,18	0,23
11	1	high	50	60	1,45	1,00		0,03	0,07
12	1	absent	15	10	0,08	0,07		0,16	0,01
13	2	low	150	80	3,98	0,44		0,60	0,10

*Mandard system modified by Ryan; TRG 0-1, Good Responder; TRG 2-3, Poor responder.
